# Dexamethasone Restores the Repressive Effect of Tumor Necrosis Factor-α on Follicular Growth and E_2_ Secretion During the In Vitro Culture of Preantral Follicles

**DOI:** 10.1007/s43032-021-00521-6

**Published:** 2021-04-06

**Authors:** Que Wu, Tingwei Zhuang, Zhiling Li

**Affiliations:** 1 Reproductive Center, The First Affiliated Hospital of Shantou University Medical College, Shantou University, Shantou City, 515041 Guangdong China; 2 The First People Hospital of Foshan, Foshan City, 528000 Guangdong China

**Keywords:** Dexamethasone, TNF-α, In vitro culture, Polycystic ovary syndrome, Preantral follicle

## Abstract

As a proinflammatory cytokine, tumor necrosis factor-α (TNF-α) is central to the female reproductive tract and affects various phases of follicular development and uterine cycles. High levels of TNF-α play a vital role in the pathogenesis of polycystic ovary syndrome (PCOS) in patients. Clinicians know that dexamethasone can inhibit the induction of androgen by suppressing the adrenal glands which improves the status of the endocrine system in PCOS patients. We hypothesize that dexamethasone has much more functionality and can exert a therapeutic effect by antagonizing TNF-α. We added TNF-α to the follicular culture medium to simulate the high TNF-α levels observed in the endocrine environment of PCOS patients. Dexamethasone was added to the medium to determine if it could counteract the inhibitory effect of TNF-α on follicular growth and 17β-estradiol (E_2_) secretion. Follicular diameter, E_2_ concentration, follicle survival, antral-like cavity formation, and ovulation were measured to assess the effects of dexamethasone. In our work, TNF-α inhibited in vitro follicular growth and E_2_ secretion in a dose-dependent manner. Based on the results of the present research, we concluded that the addition of dexamethasone partially counteracts the repressive effect of TNF-α on follicle growth and E_2_ secretion during in vitro culture of the preantral follicles of mice. Thus, the findings in this paper suggest that dexamethasone may act as a therapy by counteracting the effects of TNF-α in PCOS patients. These results provide a new foundation for exploring the treatment of PCOS patients with dexamethasone.

## Background

As an inflammation-associated cytokine, tumor necrosis factor-α (TNF-α) is connected to various biological processes, including inflammation, cell apoptosis, and necrosis [1]. It also has substantial effects on the female reproductive tract including follicular growth, ovulation, luteal formation, atresia, and exfoliation of the endometrium [2]. TNF-α is mainly secreted by various inflammatory cells and is also secreted by and can regulate the function of ovarian cells [3]. TNF-α can be detected in granulosa cells, thecal cells, and oocytes at different phases of ovarian follicular development in mice [4] as well as in the different phases of bovine follicular growth. Excessive secretion of TNF-α inhibits cell viability and promotes apoptosis in ovarian tissue [5].

Polycystic ovary syndrome (PCOS) is a complex endocrinopathy in women of reproductive age with a prevalence of approximately 6–8% women worldwide [6]. Insulin resistance (IR), hyperandrogenism (HA), and obesity (Ob) are three principle features of PCOS. Although the major cause of this endocrine dysfunction is still unclear, there is a widely accepted association between PCOS and chronic inflammation. Thus, it is widely accepted that TNF-α is essential in the pathogenesis of PCOS because it promotes IR, which causes HA and therefore participates in follicular development [7]. Additionally, TNF-α is significantly higher in the serum and follicular fluid of PCOS patients compared to non-PCOS patients [7–12]. Based on these studies, exogenous TNF-α can be added to culture systems to simulate the saturated TNF-α endocrine environment in PCOS patients and explore the influence of different concentrations of TNF-α on follicle growth and hormone secretion [5].

Clinicians know that treatment with dexamethasone is beneficial to PCOS patients. The combination of dexamethasone and clomiphene citrate has been observed to increase ovulation and pregnancy rates in PCOS patients that are resistant to clomiphene citrate [13, 14]. The literature shows that treatment with glucocorticoids can reduce androgen levels in the serum and follicular fluid of PCOS patients thereby improving their already compromised endocrine environment [15]. It has been documented that glucocorticoids, such as dexamethasone, can disrupt and ultimately hinder apoptosis induced by TNF-α in human granulosa cells [16]. We hypothesized that dexamethasone could improve follicle viability by counteracting this repressive effect of TNF-α. Consequently, the establishment of an in vitro follicle culture system offers a feasible way to study follicle development and hormone secretion. In this paper, TNF-α was added to the culture medium to simulate the high TNF-α levels observed in the endocrine environment in PCOS patients. Dexamethasone was then added to test its effects on the repressive action of TNF-α on the viability and growth of mice follicles.

## Methods

### Animals

This study was approved by the Experimental Animal Ethics Committee of our organization (SUMC2014-014). Treatment of the mice complied with the Guide for the Care and Use of Laboratory Animals by the National Institute of Health (NIH Publication No. 85–23, revised 1996) and the rules of the National Animal Protection of China. All female Kunming mice used in the present study were in the preadolescence stage (PND12-14). They were purchased from the Animal Center of Shantou University Medical College.

### Reagents

HEPES MEM (catalog no. 32360034), α-minimal essential medium (MEM, catalog no. 41090101), and insulin-transferrin-selenium mix (ITS, catalog no. 41400-045) were obtained from Gibco, Invitrogen. Ten percent fetal bovine serum (FBS) was obtained from Thermo Fisher Scientific (Waltham, MA, USA). Human pituitary follicle-stimulating hormone (FSH) was kindly donated by the Reproductive Center at the First Affiliated Hospital of Shantou University Medical College. TNF-α (catalog no.96-300-01A-10) and epidermal growth factor (EGF, catalog no.96-AF-100-15-1000) were purchased from PeproTech. Human chorionic gonadotropin (HCG, catalog no.93-4778-1000) was obtained from BioVision. Dexamethasone (catalog no. T6674) and mineral oil (catalog no. M5310) were obtained from Sigma. Human tubal fluid (HTF) was obtained from Sage Science.

### Separation of the Preantral Follicles

We isolated mouse preantral follicles from ovaries as described previously [17–19]. Prepubertal female mice were euthanized by cervical dislocation and then sterilized with 75% ethanol. Both ovaries were then removed under aseptic conditions. Under an optical microscope, the ovaries were mechanically dissected, and then the preantral follicles were mechanically isolated from the ovaries using two 1-mL syringes.

The preantral follicles were washed twice with a follicular separation solution {HEPES-MEM+10% fetal bovine serum (FBS)}, which was followed by one wash with a culture medium, and then they were grown as droplet cultures. The follicles selected for inclusion in the present study had the following characteristics: (1) the diameter of the follicles ranged from 120 to 150 μm, (2) viable oocytes were clearly in the center of the follicle, and (3) the basement membranes were intact and had attached theca cells [20, 21]. All steps were carried out in a console that was maintained at a constant temperature of 37°C.

### In Vitro Growth of the Preantral Follicles

**Fig. 1:**
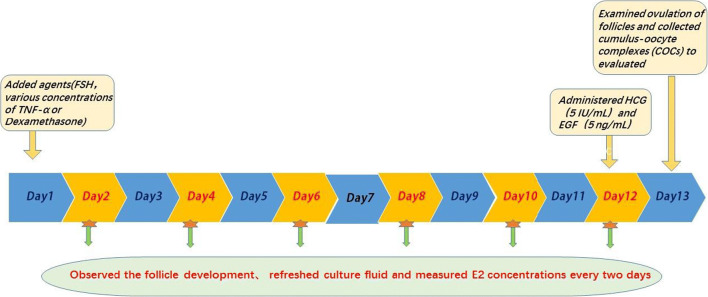
Timeline of the in vitro culture of preantral follicles

All preantral follicles were grown in vitro in a culture medium containing α-MEM, 10% FBS, and 10 μL/mL ITS (Fig. 1) [17–19]. Prior to use, the culture medium was sterilized using a 0.22-um pore filter.

The follicles were cultivated separately in 30 μL droplets of culture medium under oil in 35-mm Petri dishes (Falcon, Becton Dickinson, Belgium) in an incubator set to 37°C with 5% CO_2_ in the air. The follicles were grown in vitro for 13 days, and the development of the follicles was observed every 2 days under an inverted microscope and photographed using a camera system (Olympus CKX41) (Fig. 1). Every 2 days, when the culture fluid was renewed, the 2-day old culture fluid was collected to measure the concentration of E_2_ (Fig. 1). The diameter of each follicle was measured on the photographs using the Mshot Image Analysis System.

On day 12, 5 IU/mL of HCG and 5 ng/mL of EGF were added to the culture medium to induce ovulation and meiotic resumption (Fig. 1). The ovulation of the follicles was examined on day 13, at 20 h after the addition of the HCG [22, 23]. The collected cumulus-oocyte complexes (COCs) were repeatedly pipetted a minimum of three times, with 80 IU/mL hyaluronidase to remove the surrounding cumulus cells (Fig. 1). Once the oocytes were released, their maturation was evaluated and divided into three phases: (1) germinal vesicle (GV) phase, (2) metaphase I (MI), and (3) metaphase II (MII) [24].

The following three sections describe the changes to the abovementioned base procedure for the three control and experimental groups.

#### The Effects of FSH

**Fig. 2:**
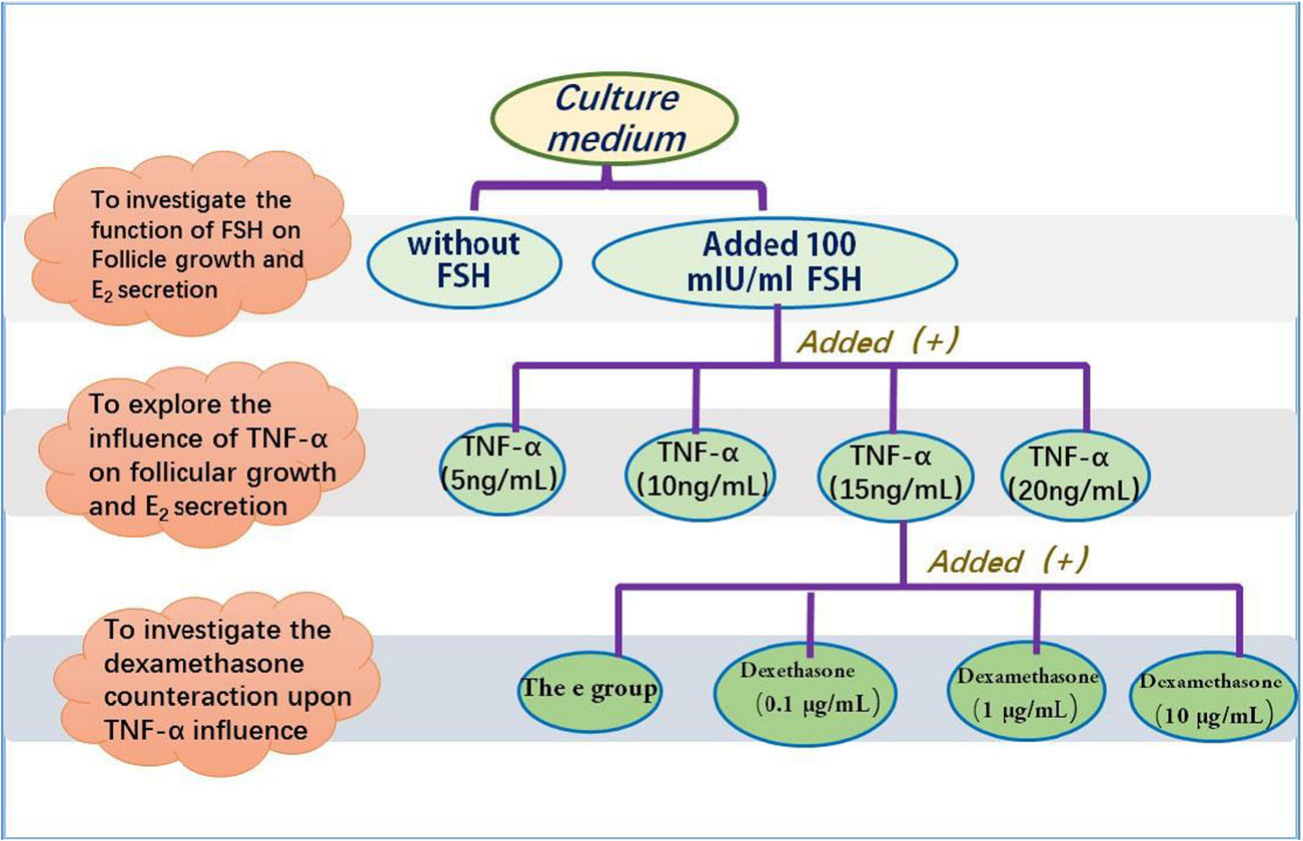
An illustration of the control and experimental groups to determine the effects of FSH, TNF-α, dexamethasone, and their interactions on follicle growth and E2 secretion

To determine the effects of FSH on follicle growth and E_2_ secretion, on day 1, the culture medium of a subset of samples was supplemented with FSH for the positive control (FSH+) and those without FSH served as the negative control (FSH−) (Figs. 1 and 2). To meet the minimum effective concentration of FSH needed to promote follicular growth [17], 100 mIU/mL of FSH was added to the culture medium of the positive controls and to additional samples that were subsequently used to explore the influence of TNF-α on follicular growth and E_2_ secretion and the impact of dexamethasone on the effects of TNF-α (Fig. 2).

#### The Effects of TNF-α on Follicle Growth and E_2_ Secretion

Previous studies demonstrated that adding TNF-α to the culture medium that achieved concentrations higher than 10 ng/mL reduced the survival of follicles of cows[5]. Consequently, on day 1 of the present study, TNF-α was added to the culture media already containing 100 mIU/mL of FSH, to achieve concentrations of 5 ng/mL (T_5_), 10 ng/mL (T1_0_), 15 ng/mL (T_15_), and 20 ng/mL (T_20_), respectively (Figs. 1 and 2). Prior to use, the TNF-α was dissolved in ultrapure water to a concentration of 10 μg/mL, aliquoted, and stored at −80°C. We referred to previous studies [18, 25] on the influence of TNF-α on ovaries, follicles, and follicular cells.

#### Impact of Dexamethasone on the Effects of TNF-α

On day 1, dexamethasone was added to samples already containing 100 mIU/mL of FSH and 15 ng/mL TNF-α, to determine its impact on the effects of *TNF-α* on follicle growth and E_2_ secretion (Figs. 1 and 2). The dexamethasone was dissolved in absolute ethyl alcohol at 1 mg/mL for storage. Working concentrations of dexamethasone were 0.1 μg/mL (D_0.1_), 1 μg/mL (D_1_), and 10 μg/mL (D_10_). The final concentration of absolute ethyl alcohol in the culture fluid did not exceed 0.1%.

On day 1, to achieve a concentration of 0.1% ethanol in the culture fluid, ethanol was added to a subset of samples already containing 100 mIU/mL of FSH and 15 ng/mL TNF-α (Fig. 2). This was done to control (e-group) for any effects of the ethanol on follicle growth and E_2_ secretion (Fig. 2).

#### Assessing the Influences of FSH, TNF-α, and Dexamethasone

To assess the influences of FSH, TNF-α, and dexamethasone on follicle growth, the following variables were observed and measured: (1) follicle diameter, (2) the concentration of E_2_ in the medium, (3) follicle survival, (4) antral-like cavity formation, and (5) ovulation. Furthermore, the first polar body (PB) excretion ratio was used as an indicator of oocyte nuclear maturation. It was calculated by dividing the number of MII oocytes by the number of ovulated follicles.

### Assessment of Follicle Survival

The morphological changes were evaluated in accordance with previous studies [26, 27], and the follicles were assigned to a growth phase as described below. Follicles were in the follicular phase when they maintained a spherical structure, theca cells attached to the culture dish, and the basement membrane restricted the proliferation of the granulosa cells. The outgrowing phase occurs when the granulosa cells proliferate significantly and was observed on days 4 to 6 when the granulosa cells grew beyond the basement membrane and the volume of the follicles became larger than before. Lastly, the follicles are in the antral phase when the follicular fluid filled an area around the granulosa cell layer indicating the formation of an antral-like cavity. The follicle became degenerated when the oocyte turned dark or became broken and granulated [28]. In addition, we defined and calculated the following three numerical indexes: the surviving follicle fraction, which is calculated by dividing the number of surviving follicles by the total number of follicles; the antral-like cavity formation percentage, which was calculated by dividing the number of antral follicles by the total number of follicles; and the ovulation fraction, which was calculated by dividing the number of ovulated follicles by the number of surviving follicles.

### 17β-estradiol Detection

The culture fluid was refreshed every 2 days. The collected medium was pooled and frozen at −80°C until an assay of its E_2_ level was conducted. Estradiol EIA kits from Cayman (catalog number. 582251) were used according to the manufacturer’s instructions, to measure the E_2_ concentrations in the recovered culture fluid. The culture fluid of non-viable follicles was excluded from the analyses.

### Statistical Analysis

All tests were repeated a minimum of three times. One-way ANOVA was used to evaluate the differences between means using a post hoc test. Differences in percentages were compared using a chi-square test using the software SPSS19.0. Data were expressed as the mean ± SD. A *P* < 0.05 was considered statistically significant.

## Results

### Follicle Growth and E_2_ Secretion in the Presence and Absence of FSH

**Fig. 3:**
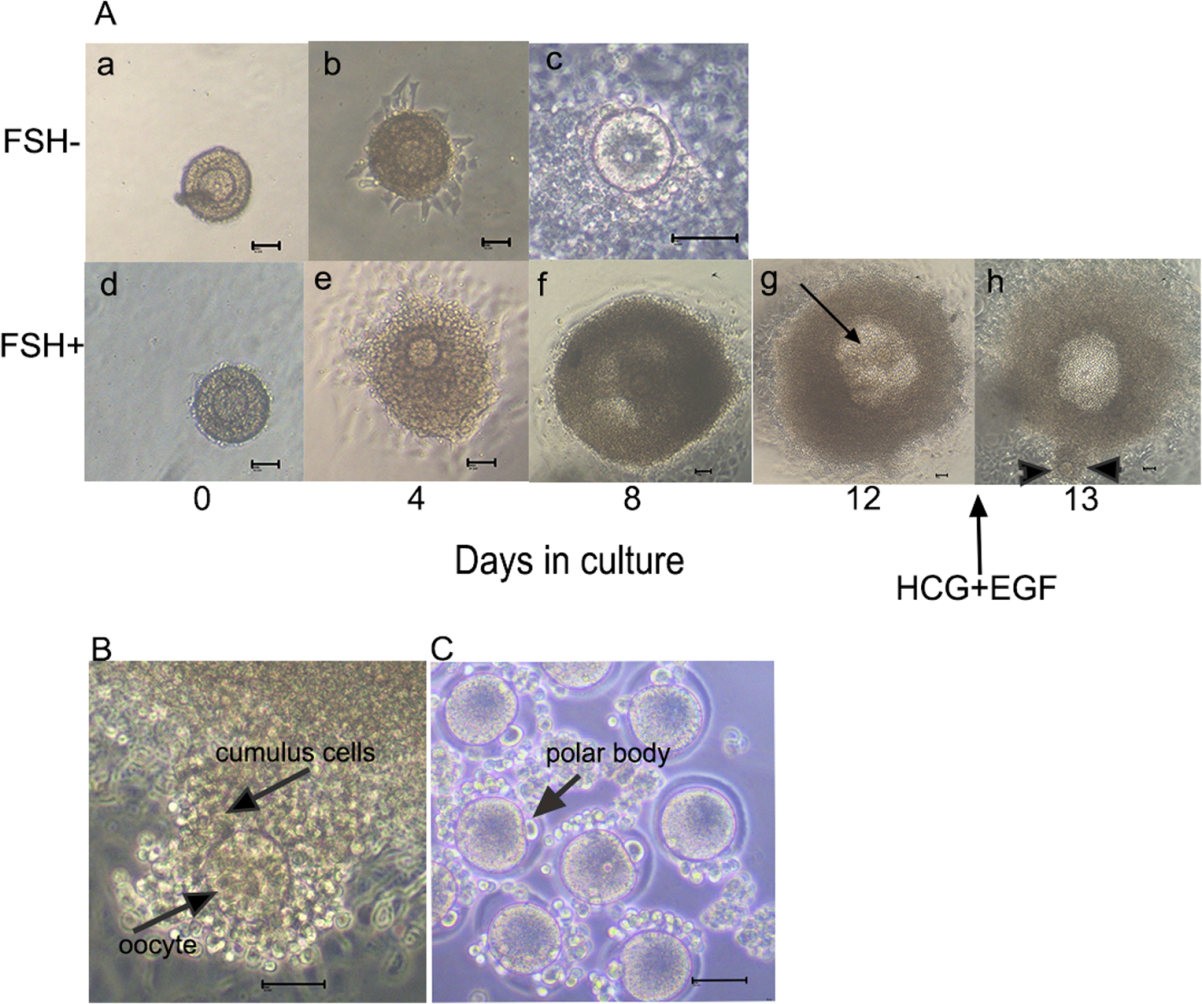
Microphotographs showing in vitro growth of mouse preantral follicles with and without FSH. **A** Examples of follicular growth with or without FSH (100mIU/mL). Most of the follicles in the FSH− group were degraded and broken (c), but follicles treated with FSH became larger and formed antral-like cavities after 8 to 12 days in culture. Follicles formed obvious antral-like cavities after 12 days in culture. Arrowhead shows an antral-like cavity (g). Supplementation with 5 IU/mL of HCG and 5ng/mL of EGF causes the COC to be released from the follicle after 20 h. Dual arrows refer to released COC (h) (bar =50μm). **B** A picture of an ovulated COC with extension of the cumulus (bar =50μm).**C** In the FSH+ group, using 80IU/mL of hyaluronidase to remove surrounding cumulus cells, we can observe a group of metaphase II oocytes (bar =50μm)

**Fig. 4:**
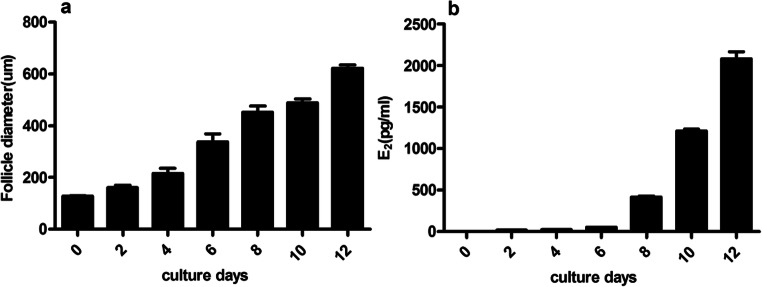
Influence of FSH on follicle diameter and E_2_ secretion. **a** Diameter changes of follicles in the FSH+ group during in vitro growth. **b** Changes of E_2_ levels in culture medium in the FSH+ group during in vitro growth

Over a 12-day period in the follicle culture system, the vast majority of follicles survived and grew larger. Additionally, the follicles traversed the preantral, antral, and preovulatory phases, which is consistent with in vivo growth patterns. The survival of follicles treated with FSH was similar in every independent experiment. The addition of FSH stimulated the growth of follicles and increased the secretion of E_2_. On day 12, the surviving follicle fraction between the FSH− and FSH+ groups were 9.3% and 92.8%, respectively. The percentage of follicles forming the antral-like cavities in the FSH− group was almost 0%, whereas it reached 63.8% in the FSH+ group. The ovulation fraction was 9.1% on the 13th day after HCG stimulation in the FSH− group but attained 72.4% in the FSH+ group. With the extension of the cumulus, each COC had mucification (Fig. 3B), and 60% of the observed oocytes were at metaphase II. With the addition of FSH, the follicular diameter and E_2_ levels in the media rose gradually during culture and dramatically increased on day 12 compared with day 2 (Fig. 4).

### Influence of TNF-α on Follicular Growth and E_2_ Secretion

**Fig. 5:**
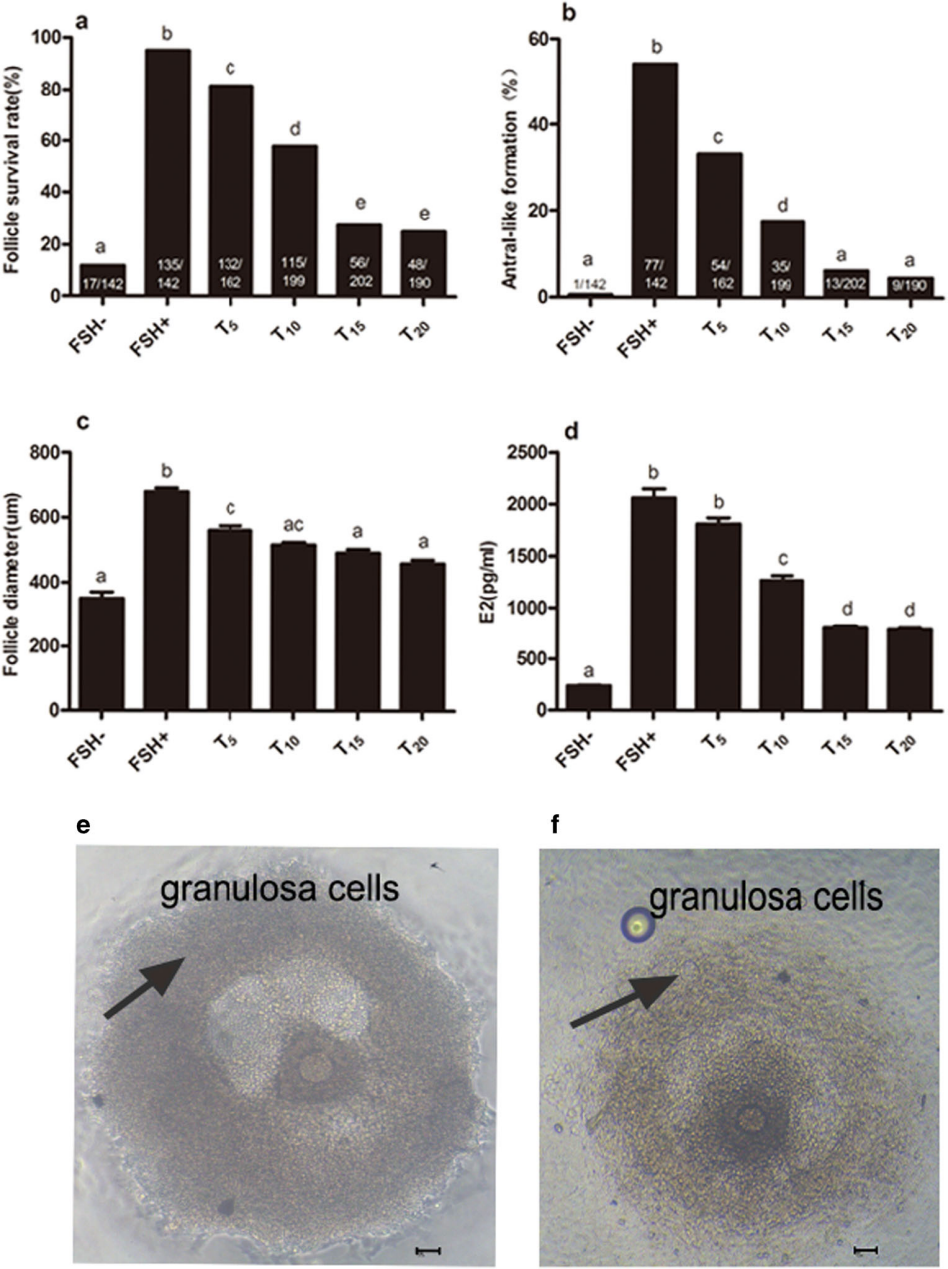
Influence of TNF-α on follicle growth and E_2_ secretion. Follicles were treated with 5, 10, 15, and 20ng/mL TNF-α, and follicle development was assessed under an inverted microscope. **A** Influence of different doses of TNF-α on follicular survival after 12 days in culture. Numbers inside of the bars demonstrate surviving follicles/total follicles. **B** Influence of different doses of TNF-α on follicular antral-like cavity formation rates after 12 days in culture. Numbers inside of the bars demonstrate follicles with an antral-like cavity/total follicles. **C** Influence of different doses of TNF-α on follicular diameter after 12 days in culture. Data are represented as means ±SD. **D** Influence of different doses of TNF-α on follicular E_2_ secretion after 12 days in culture. Data are represented as means ±SD. All tests comprised fewer than 3 independent tests. The FSH− group without FSH and TNF-α served as negative controls. The different letters on the bars indicate statistical significance (*P<0.05*). **E** In the FSH+ group, granular cell layers grew more densely (bar =50μm). **F** In the T_15_ group, granular cell layers were sparse (bar =50μm)

TNF-α inhibited the growth of the preantral follicles and E_2_ secretion in a dose-dependent manner (Fig. 5). The addition of TNF-α significantly reduced the proliferation of the granulosa cell layer and, in particular, the mural granulosa cell layer, which was consistent with a decrease in E_2_ secretion. TNF-α at 10 ng/mL, 15 ng/mL, and 20 ng/mL all resulted in a small follicle diameter and low E_2_ secretion on day 12. Furthermore, TNF-α also reduced the survival and antral-like formation of follicles on day 12 but did not affect the first PB rate. As shown in Table 1, 47.7%, 23.8%, and 21.6% of the total number of follicles ovulated when the dose of TNF-α was 10 ng/mL, 15 ng/mL, or 20 ng/mL, respectively. These percentages show a reduction in the proportion of ovulated follicles compared to the 67% observed for the FSH+ group. However, 82.6%, 85.7%, and 85.4% of all surviving follicles ovulated, respectively. These percentages show a slight increase compared with the 70.4% observed for the FSH+ group. These differences are due to the addition of TNF-α which reduced the survival of the follicles but increased the ovulation rate among all surviving follicles.. In summary, TNF-α (15 ng/mL) inhibits the growth of follicles and the secretion of E_2_. As such the following experiments used TNF-α at a concentration of 15 ng/mL.

**Table 1:** Influence of TNF-α on ovulation and meiotic resumption during the in vitro growth of follicles

Treatment	Number of ovulated follicles/total follicles (%)	Number of ovulated follicles/surviving follicles (%)	Number of MII oocytes/ovulated follicles (%)
FSH−	4/142 (2.8)a	4/17 (23.5)^a^	2/4 (50) ^**a**^
FSH+	95/142 (66.9)^b^	95/135 (70.4)^b^	61/95 (64.2)^a^
T_5_	115/162 (71)^b^	115/132 (87.1)^c^	68/115 (59.1)^a^
T_10_	95/199 (47.7)^c^	95/115 (82.6)^bc^	62/95 (65.3)^a^
T_15_	48/202 (23.8)^d^	48/56 (85.7)^bc^	31/48 (64.6)^a^
T_20_	41/190 (21.6)^d^	41/48 (85.4)^bc^	24/41 (58.5)^a^

Different superscript letters(^a–d^) in the same column represent significant differences; *P* <0.05

### Dexamethasone Rescues TNF-α Repression of Follicular Growth and E_2_ Secretion

**Fig. 6:**
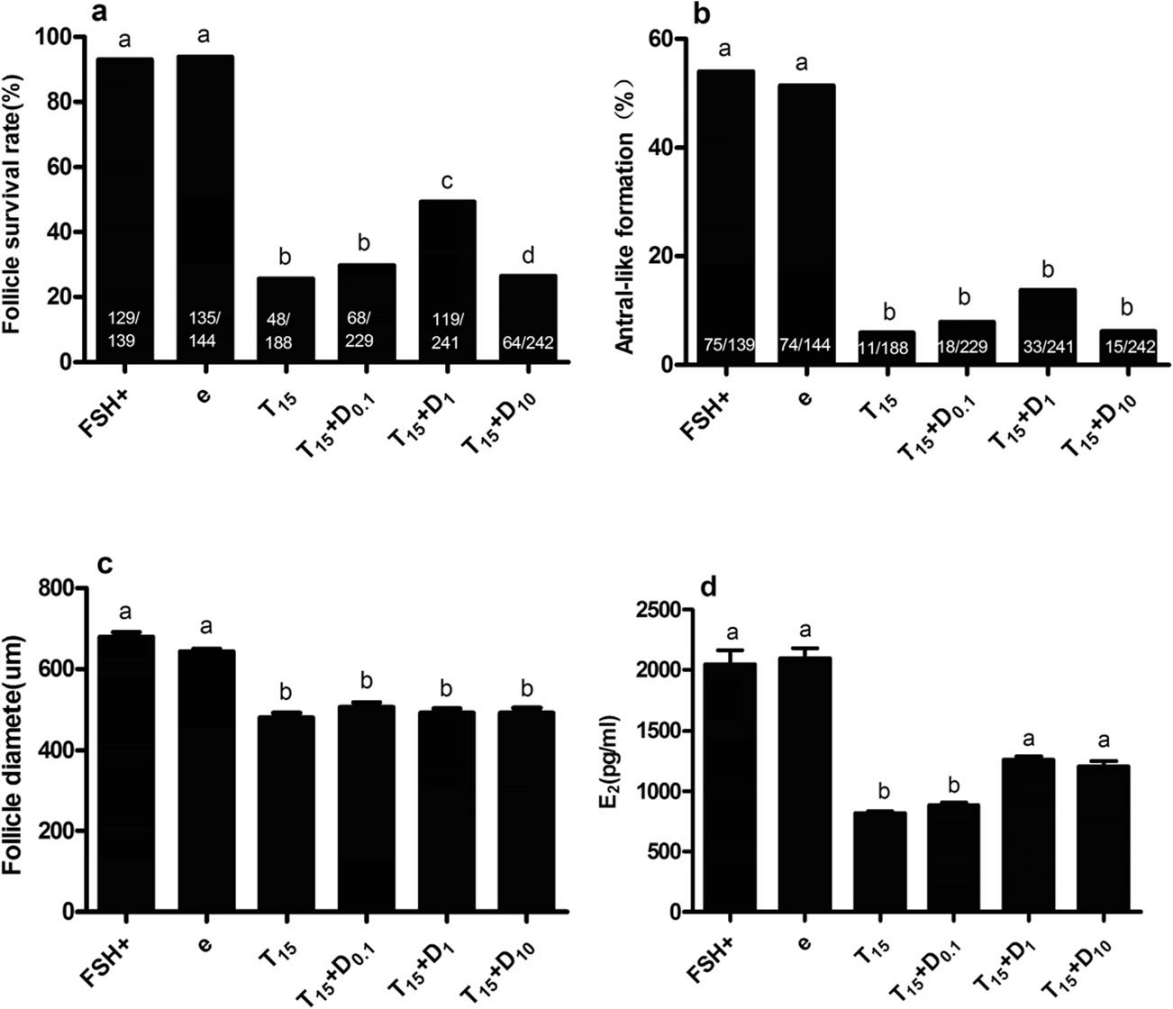
Dexamethasone restores the inhibitory effect of TNF-α on follicular growth and E_2_ secretion. Follicles were treated with 0.1, 1, or 10μg/mL of dexamethasone in the presence of 15ng/mL of TNF-α. **a** The influence of various compounds on follicular survival after 12 days in culture. Numbers inside of the bars demonstrate surviving follicles/total follicles. **b** The influence of various compounds on follicular antral-like cavity formation after 12 days in culture. Numbers inside of the bars demonstrate follicles with an antral-like cavity/total follicles. **c** The influence of various compounds on follicular diameter after 12 days in culture. Data are represented as means ±SD. **d** Influence of various compounds on follicular E_2_ secretion after 12 days in culture. Data are represented as means ±SD. All tests comprised fewer than 3 independent tests. Follicles were treated with absolute ethyl alcohol as the only vehicle control (e group). The different letters on the bars indicate statistical significance (*P<0.05*)

Dexamethasone partially counteracted the effects of TNF-α in a dose-dependent manner (Fig. 6). TNF-α inhibited follicular growth and E_2_ secretion. The survival rate of follicles on day 12 decreased from 92.8 to 25.5%, and the concentration of E_2_ in the culture medium was significantly reduced from 2046 ± 332.9 to 815.6 ± 54 pg/mL (*P* < 0.05). However, when 1 μg/mL of dexamethasone was added to the cultures, the survival rate of the follicles increased from 25.5 to 49.4% and the concentration of E_2_ in the culture medium increased from 815.6 ± 54 to 1258 ± 89.8 pg/mL (*P* < 0.05) on day 12. There was no observed change in either follicle diameter or antral-like cavity formation with the addition of dexamethasone. These data and results indicate that dexamethasone counteracts the inhibitory effects of TNF-α. The total number of follicles that ovulated in the T15, T15+D0.1, T15+D1, and T15+D10 groups was 22.3%, 25.3%, 43.2%, and 23.1%, respectively, whereas the percentages of the surviving follicles that ovulated was 87.5%, 85.3%, 87.4%, and 87.5%, respectively (Table 2). The difference in the percentages might be because the dexamethasone increased the survival rate of the follicles over the 12-day period.

**Table 2:** Influence of dexamethasone on ovulation and meiotic resumption in the presence of 15 ng/mL of TNF-α

Treatment	Number of ovulated follicles/total follicles (%)	Number of ovulated follicles/surviving follicles (%)	Number of MII oocytes/ovulated follicles (%)
FSH+	91/139 (65.5)^a^	91/129 (70.5)^ab^	60/91 (65.9)^a^
e	89/144 (61.8)^a^	89/135 (66)^b^	58/89 (65.2)^a^
T_15_	42/188 (22.3)^b^	42/48 (87.5)^abc^	26/42 (61.9)^a^
T_15_+D_0.1_	58/229 (25.3)^b^	58/68 (85.3)^abc^	38/58 (65.5)^a^
T_15_+D_1_	104/241 (43.2)^c^	104/119 (87.4)^c^	61/104 (58.7)^a^
T_15_+D_10_	56/242 (23.1)^b^	56/64 (87.5)^ac^	31/56 (55.4)^a^

Different superscript letters(^a–c^) in the same column represent significant differences; *P* <0.05

**Fig. 7:**
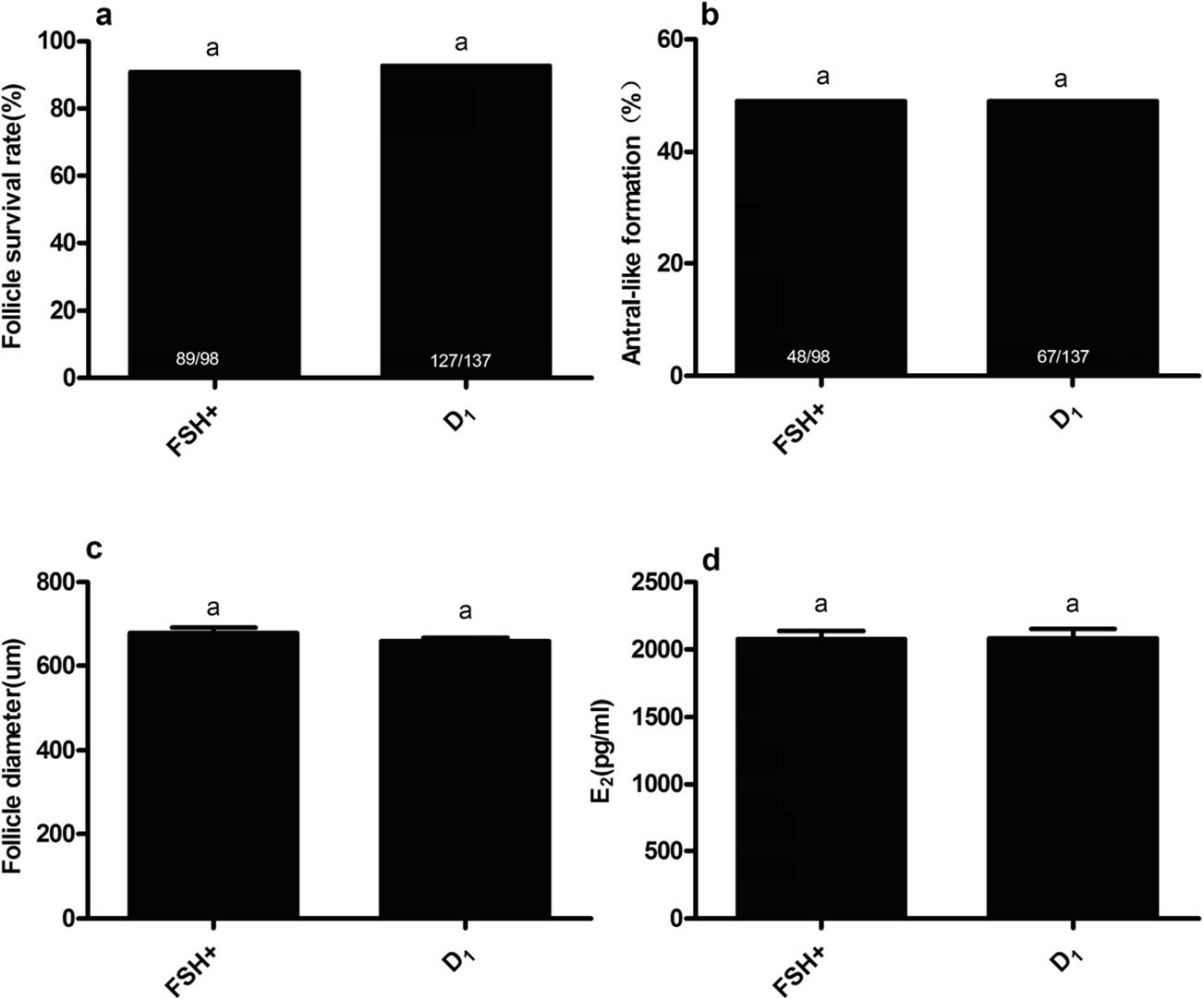
Influence of dexamethasone (1μg/mL) on follicular growth and E_2_ secretion. Follicles treated with and without 1μg/mL dexamethasone*.* **a** Influence of 1μg/mL dexamethasone on follicle survival after 12 days in culture. Numbers inside of the bars demonstrate surviving follicles/total follicles. **b** Influence of 1μg/mL dexamethasone on follicular antral-like cavity formation after 12 days in culture. Numbers inside of the bars demonstrate follicles with an antral-like cavity/total follicles. **c** Influence of 1μg/mL dexamethasone on follicular diameter after 12 days in culture. Data are represented as means ±SD. **d** Influence of 1μg/mL dexamethasone on follicular E_2_ secretion after 12 days in culture. Data are represented as means ±SD. All tests comprised fewer than 3 independent tests. The *e* group was treated with absolute ethyl alcohol only. Controls were treated with FSH only. Different letters on the bars indicate statistical significance (*P<0.05*)

Granulosa cells of the surviving follicles in the TNF-α group grew only sparsely. At a concentration of 1 μg/ml, the dexamethasone promoted the proliferation of the granulosa cells, which are responsible for the secretion of E_2_ and explains the indirect effect of dexamethasone on E_2_ secretion. In addition, no germinal vesicle was observed in the oocytes of each experimental group, and there were no statistically significant differences in the PB rates among the groups. There was no evidence in the present study that dexamethasone and TNF-α had an effect on nuclear maturation. The addition of 1 μg/mL dexamethasone alone had no effect on follicle growth and E_2_ secretion (Fig. 7; Table 3)**.**

**Table 3:** Influence of dexamethasone (1μg/mL) on ovulation and meiotic resumption during follicle in vitro growth

Treatment	Number of ovulated follicles/total follicles (%)	Number of ovulated follicles/surviving follicles (%)	Number of MII oocytes/ovulated follicles (%)
FSH+	61/98 (62.2)^a^	61/89 (68.5)^a^	42/61 (68.9)^a^
D_1_	86/137 (62.8)^a^	86/127 (67.7)^a^	56/86 (65.1)^a^

Different superscript letters in the same column represent significant differences; *P* <0.05

## Discussion

The present study successfully established an in vitro preantral follicle culture system. This is because the follicles traversed the preantral, antral, and preovulatory phases by day 12 of the experiment, which is consistent with the in vivo growth patterns of follicles. In addition, we proved that the addition of FSH is beneficial and essential to the in vitro development of follicles. As described in Fig. 2, we added different concentrations of TNF-α to the FSH(+)group, to explore the influence of them on follicular growth and E_2_ secretion.

Data collected from these experiments illustrate that the administration of TNF-α inhibits follicle growth and E_2_ secretion, which is consistent with previous studies [17, 18, 29, 30]. In the in vitro follicle culture system, TNF-α significantly impaired FSH-induced follicle development and E_2_ production in a dose-dependent manner. The minimum effective concentration at which TNF-α inhibits follicle growth and hormone secretion is 5 ng/mL as previously reported [18], but our results suggest that it is 15 ng/mL. This discrepancy may be due to the different experiment mouse species used, the dissolution method, the preservation conditions of the reagents, and variations in the individual operations. Granulosa cells and especially the mural granulosa cells of the follicles treated with 15 ng/mL TNF-α were lower number of cells than that treated with only FSH. TNF-α was demonstrated to impair follicle growth by mainly destroying the growth of granulosa cells and that this resulted in a reduction in the secretion of E_2_. These results are consistent with those of previous studies [31].

Hara et al. argued that TNF-α primarily affects antral and not preantral follicles [18]. However, the present study showed that TNF-α has an inhibitory effect on preantral follicle survival. Furthermore, the percentage of surviving follicles was slightly higher in the TNF-α-treated groups compared to the groups with no TNF-α added. This shows that TNF-α can increase the rate of ovulation caused by HCG. Ovulation is a complex physiological process involving a series of biochemical and physiological events [32], and a growing number of scientists consider TNF-α to be an essential regulator during ovulation through the promotion of apoptosis in follicular cells and lysis of the extracellular matrix of the follicular wall [33]. Our experimental results confirm these findings.

In addition, our results show that dexamethasone partially counteracts the inhibitory effect of TNF-α on follicle growth. From the results, the addition of dexamethasone increased the survival of TNF-α-treated follicles, especially in the T_15_ group. The addition of dexamethasone could only partially reverse the TNF-α inhibition of follicular growth and E_2_ secretion Although the diameter of the follicles did not increase, dexamethasone did promote the proliferation of granulosa cells and increased the secretion of E_2_ in the follicles treated with TNF-α. These results suggest that in a high TNF-α environment, dexamethasone can improve the levels of steroids, thereby increasing the survival of follicles. However, treatment with 1 μg/mL dexamethasone alone did not change the development of the follicles or affect steroid hormone production. The addition of dexamethasone preserves the follicular microstructure of the ovary [5]. We suspect that dexamethasone alone may have an effect on the ultrastructure of follicles but that these effects are not enough to cause substantial changes in the diameter of the follicle and improve E_2_ production. These aspects require detailed research.

It is believed that hyperandrogenism in PCOS is, to some extent, due to excess production of adrenalin by the adrenal glands and that this complex of metabolites in PCOS results in therapeutic challenges [34]. It is well-known that dexamethasone can inhibit the induction of androgens through suppression of the adrenal glands resulting in an improvement in the endocrine status of PCOS patients [15, 35–37]. However, our results show that dexamethasone may be used for the treatment of PCOS because of its antagonistic effect on TNF-α. In PCOS patients, high TNF-α levels not only inhibit follicular growth but also increase insulin resistance. In adolescents with PCOS whose serum TNF-α is significantly elevated, dexamethasone may be used as an early treatment to reduce the risk of metabolic diseases and promote follicle growth. This provides a new direction for the treatment of adolescent PCOS patients. Further research is needed to determine the appropriate dosage and when to administer dexamethasone as treatment to treat adolescent PCOS patients.

## Conclusions

In our in vitro follicle culture system, TNF-α inhibited the growth of preantral follicles and inhibited the secretion of E_2_. The addition of dexamethasone partially counteracted these inhibitory effects of TNF-α. The results of the present study suggest that dexamethasone may exert a therapeutic effect because it is antagonistic to TNF-α. Based on these results, there is a new foundation from which to conduct research on the application of dexamethasone in PCOS patients with high TNF-α levels.

## Data Availability

Data sharing is not applicable to this article as no datasets were generated or analyzed during the current study.

## Abbreviations

PCOS: polycystic ovary syndrome
TNF-α: tumor necrosis factor-α
FSH: follicle stimulating hormone
E_2_: 17β-estradiol
HCG: human chorionic gonadotropin
MEM: minimal essential medium
COC: cumulus oocyte complex
PB: polar body
EGF: epidermal growth factor
